# Hepatitis C Virus Infection in Pregnancy and Children: Its Implications and Treatment Considerations with Directly Acting Antivirals: A Review

**DOI:** 10.31729/jnma.5501

**Published:** 2021-09-30

**Authors:** Ramesh Rana, Rajkumar Dangal, Yogendra Singh, Ram Bahadur Gurung, Bhim Rai, Amit Kumar Sharma

**Affiliations:** 1 Department of Medicine, Ungoofaaru Regional Hospital, Ungoofaaru, RAA Atoll, Maldives; 2 Department of Medicine, Laligurash Hospital, Talchhikhel-14, Lalitpur, Nepal; 3 Dolakha Hospital, Kathmandu University-affiliated Hospital, Dolakha, Nepal; 4 Department of Medicine and Gastroenterology & Endoscopy Unit Endoscopy Training Center, Dhulikhel Hospital, Kathmandu University School of Medicine, Dhulikhel, Nepal; 5 West Mersea GP Practice, Mersea Island, Colchester, UK

**Keywords:** *antiviral agents*, *hepatitis C virus*, *interferon-alpha*, *pregnancy*, *review*, *transmission*, *treatment*

## Abstract

Hepatitis C virus infection is a global health problem affecting >71 million people worldwide with chronic hepatitis C, 40% reproductive age group, and 8% pregnant women. Intravenous drug abuse, multi-transfusions are major risk factors in adults, while vertical transmission in pediatric population. It presents as a chronic liver disease, has higher risk of liver cirrhosis and even progression to hepatocellular carcinoma. Proper screening of high-risk populations including pregnancy is recommended. All diagnosed chronic hepatitis C cases should be treated with directly acting antivirals including pre-conception. This would reduce the disease burden, vertical transmission, and disability associated. However, no directly acting antivirals regimens recommendation till date due to lack of evidence on adverse fetal outcomes and are concerned about the pharmacokinetic effect regarding physiological changes during pregnancy. Therefore, in this review, we have tried to explore the possible use of directly acting antivirals regimens and their safety issues during pregnancy, and possible consideration of few pan-genotypic regimens in the late second and early third trimester. This would not only prevent vertical transmission and decrease disease burden but also help to meet the World Health Organisation 2030 target of hepatitis C virus elimination as a major public health problem.

## INTRODUCTION

Hepatitis C virus (HCV), a single-stranded RNA virus, has seven genotypes (GT), usually present with chronic liver disease.^1-3^ GT 1-3 are globally prevalent while GTs 4, 5, and 6 are localized in Northern Africa, South Africa, and Asia, respectively.^2,4-6^

Approximately 170 million people are HCV infected globally, 8% of pregnant women, pose a significant risk in development of liver cirrhosis (27%) and hepatocellular carcinoma (25%).^3,7-13^ Particularly, women have a higher infections risk; about 40% of reproductive age group.^14-16^Around 2-8% mother-to-child transmission (MTCT) risk from HCV monoinfected women,^7^ studies suggest HCV associated with low gestational weight, diabetes, hypertension, and preterm birth.^9,14,17^

Controversies exist regarding use of HCV antivirals (DAAs) and their safety issues in pregnancy; therefore, we tried to discuss their implications during pregnancy, and important judgment has to be made given mother and child health reducing HCV burden and associated complications.

## EPIDEMIOLOGY

The HCV infection has been systematically studied and characterized in North America and Europe, while in other parts of the world this has not received equivalent attention as an important public health problem.^18^ WHO estimates 71 million chronically infected HCV cases and 399000 HCV related deaths in the year 2016.^11,19-20^ Therefore, World Health Assembly endorsed the global health sector strategy for the elimination of viral hepatitis as a public health threat by 2030 (90% reduction in new infections and 65% mortality).^21^

China has more HCV cases than combined Europe and America. Most countries have prevalence rates from 1 to 2% while Pakistan, India, central Africa, Egypt, and Russia have a relatively high prevalence in terms of absolute numbers.^9,17,18,22^ Among whom Egypt and Pakistan have the highest prevalence of HCV infections in the world, estimated nationally at 14.7%.^23-25^ Overall, HCV prevalence among pregnant women ranged between 5-15%, blood donors (5-25%),multi-transfused patients (10-55%), dialysis patients (5090%), high-risk populations (10% and 85%), and other general population (0-40%).^23,26^

Globally 2.1 million children under 15 years of age are living with chronic HCV infection. Vertical transmission remains the main cause. Pakistan (25%) and Egypt (up to 50%) have the highest estimate of HCV-infected children less than five years of age among whom vertical transmission remains the main cause.^9^

## RISK FACTORS

Hepatitis C is primarily transmitted through direct exposure to blood. Mucous membrane blood exposures can also result in transmission. Additionally, it can be detected in saliva, semen, breast milk, and other body fluids; however, these body fluids are not believed to be efficient routes of transmission.^2^

The risk of transmission can be categorized as risk behaviors, risk exposures, risk conditions, and circumstances, and as per, screening is carried out. Risk behaviors include illicit drug abuse intranasal or injectable form, men having sex with men. Risk exposures include health care personnel or public safety workers after needlestick injury, a child born to HCV-infected mother, people with percutaneous or parenteral exposures in an unregulated setting or incarcerated, repeated history of blood transfusion or long-term hemodialysis. And, risk conditions and circumstances include HIV infection, sexually active persons, solid organ donors and recipients, unexplained chronic liver disease or chronic hepatitis.^27,17,28^

## THE NATURAL HISTORY OF HCV INFECTION IN PREGNANCY

**Figure 1:**
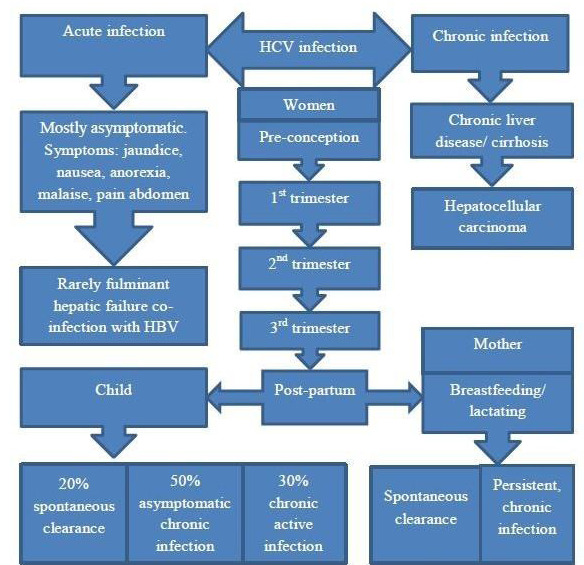
Natural history of HCV infection in pregnancy.

The natural history of acute HCV infection in pregnancy is unknown, possible natural history (Figure 1).

In women with chronic HCV infection, approximately two-thirds of women experience an increase in HCV RNA viral load, usually in the first and third trimester, peaking in the third trimester, whereas decreased in postpartum. In contrast, the hepatic transaminase levels declined in the first and third trimesters, while increased after delivery, and later normalize in 77% of women.^24,28-29^ Studies reported spontaneous clearance or undetectable HCV RNA in up to 10% of postpartum women. A study from Egypt showed a 25% spontaneous resolution and found a strong association with favorable IL 28B allele.^9,24,30-31^ The downregulation of immune responses, particularly T cell-mediated reactivity prevents maternal immune aggression against the fetus, and impaired cellular response results in decreased immune-mediated liver damage whereas favoring viral replication during pregnancy. On the contrary, T cell-mediated cytotoxicity against HCV epitopes in the postpartum period results decline in viral burden and rebound of liver injury instead.^29^ Therefore, periodic HCV RNA evaluation should be performed 9-12 months post-delivery to assess the spontaneous clearance before considering DAA treatment.^32^ Meta-analysis results demonstrated HCV infected pregnant women have a higher incidence of intrahepatic cholestasis compared to non-infected pregnant women. Additionally, those HCV-infected pregnant women who have intrahepatic cholestasis of pregnancy and higher viral loads have a higher rate of maternal and fetal adverse outcomes.^24,33,34^ Therefore, this indicates close specialties monitoring during pregnancy. Pregnant women with cirrhosis have increased the risk of poor maternal outcomes such as preeclampsia, cesarean section, hemorrhagic complication, and even death; and neonatal outcomes such as preterm delivery (60%), low birth weight, and even neonatal death. However, these perinatal outcomes risk has been confounded by associated comorbid conditions like substance use.^28,35,36^

Approximately 20% (11-25%) of children borne from HCV-infected mothers may have spontaneous remission but decreased to 10% in the case of HIV coinfection (PLHA). The remaining eighty percentage may present with asymptomatic chronic infection (50%) and active chronic infection (30%).^37,38^ The spontaneous viral clearances of HCV infected children have an association with IL-28B polymorphism and genotype 3 HCV infection. Further, it usually occurs within 6 years of life.^29,39^

## MOTHER-TO-CHILD TRANSMISSION (MTCT) OF HCV INFECTION

Globally, the vertical transmission ranges from 6-11% and the risk increases with higher viral load, illicit drugs use, and HIV co-infection.^9,26^ However, mode of delivery (cesarean section versus vaginal), breastfeeding, and viral genotypedo not correlate with risk of vertical transmission.^26^ A recent meta-analysis established an MTCT rate of5.8% in viremic women, and 10.8%-19.4% in HIV co-infected women, especially HIV-1 coinfection.^31,38,40^ It is lower in the United States (0.2%) and Europe (0.4%) but higher in Africa or some developing nations (12-15%).^8,29,41^ Although HCV transmissionis related toHCV viralload,the transmission may increase in people with HIV co-infection with similar levels of viral load; however, viral loads <10^5^ copies/mL were less likely to transmit the disease even in HIV coinfection. Maternal injecting drug use may be a risk factor for HCV transmission.^28,39^ Prolonged rupture of membranes for >6 hours and invasive fetal monitoring use may increase the risk of transmission; however, there are inconsistent findings in the literature. Moreover, an episiotomy may serve as a risk factor for MTCT. Breastfeeding is not a risk for MTCT and is not eluded; nevertheless, cracked or bleeding nipples or HIV co-infection is not a recommendation for breastfeeding. Case series demonstrated the vertical transmission of HCV to twins may be discordant. An elective cesarean section could be recommended for HCV twin pregnancy to avoid premature rupture of the membrane and to lower risk to the second twin.^8,12,28,32,42^ A few studies have revealed twofold higher perinatal transmission in females compared with males, indicating genetic or hormonal factors may affect the susceptibility to HCV infections.^39^

Apart from HCV infection, the risk of MTCT for HIV without HAART is 17-25% and that for HBV without prophylaxis is 30-40%, which is significantly reduced to 1-2% with HAART in HIV and 3-4% with HBV prophylaxis. Additionally, the maternal higher viral load had a significant risk for MTCT.^43^ Similarly, maternal viremia has a significant role in vertical transmission in HCV as well, a mother with undetectable plasma HCV-RNA levels barely transmits the disease.^44^ Therefore, treating HCV infection during pregnancy would not only cure the disease in mothers but also lower the potential risk of MTCT.^4^ This concludes that the HCV treatment during pregnancy has dual beneficial perspectives for both the mother and the child.

## CLINICAL FEATURES AND DIAGNOSIS

Acute hepatitis C is caused by primary HCV infection, majority of patients are asymptomatic. but if symptomatic, then presents with malaise, anorexia, nausea, epigastric pain, fatigue, dark urine, joint pain, and jaundice.^19^ The spontaneous remission in acute infection is about 15-45% in 6 months duration whereas approximately 55-85% progress to a chronic state which may be diagnosed during screening or patients present with complications such as liver failure, cirrhosis, and hepatocellular carcinoma (HCC).^5,11,45^ In general, the infection does not impact the clinical course of pregnancy in the absence of cirrhosis; however, there are reports of prematurity, low birth weight, antepartum hemorrhage, pre-eclampsia, and microcephaly.^9,14,17,46,47^ Gestational diabetes and gestational hypertension are common in HCV carriers; however, there was a decreased risk of preeclampsia shown by the Swedish population-based cohort study.^48^ Intrahepatic cholestasis is more common in pregnant women with HCV RNA positive state, accounts for up to 20%. There is an increase in ALT/AST in the first trimester whereas decreased in the second and third trimester of pregnancy.^19,46,49^

Besides HCV infection can have extrahepatic manifestations such as cryoglobinuria, vasculitis, autoimmunity, and dermatological manifestations in any population.^46^

## DIAGNOSIS AND WHO SHOULD BE TESTED OR TIMING OF TESTING?

**Figure 2:**
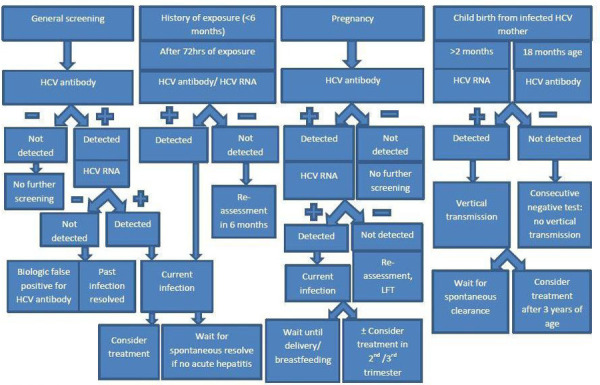
Tests recommendation in HCV infections according to cases.

Generally, every adult above 18 years of age without risk factors and all pregnant women should be screened for HCV infection. Those people with higher risk factors such as people who inject drugs, opioid use, men having sex with men, HIV/HBV infected persons, etc should be frequently screened.^24,27,50^ The general algorithm or recommendation of testing in hepatitis C infection according to the case (Figure 2).^24,45,51^

*HCV RNA can be performed in immunocompromised individuals

Basically, HCV antibody and HCV RNA test are the two tests evaluating the presence or absence and acute or chronic HCV infection. Besides, increased ALT/AST levels indicate the active infections or hepatitis state; therefore, liver function test, prothrombin time and INR, renal function test, and complete blood counts should be evaluated. Ultrasonography, safe in pregnancy, plays a prime role in evaluating chronic liver disease state andthe occurrence of HCC. Further, liver elastography and computed tomography scan can help to assess the presence or absence of cirrhosis and HCC; however, these are reserved investigations during pregnancy due to the potential risk of radiation hazards.^24,45^ In immunocompromised patients with a high risk of HCV infection, despite negative HCV antibody, confirmatory HCV RNA should be performed. Generally, genotype testing is not recommended for the treatment of naïve patients due to the availability of pan-genotypic DAAs, but there is strong guidelines recommendation while treating treatment experience or treatment failure cases.^24^ Additionally, HCV core antigen detection in blood also indicates active HCV infection; however, it lacks sensitivity. In addition, HCV RNA, a confirmatory test should be performed in HCV antibody positive and negative HCV core antigen test to exclude a false-negative core antigen result.^52^

In the case of a newborn child, as the anti-HCV crosses the placental barrier, can be detected in the neonates, and may persist up to 18 months of life. Therefore, the HCV antibody test is recommended only after 18 months of age. Whereas, the HCV RNA can be performed as early as two months of age; still, a prior test may have a falsely negative result. Ninety percentage of HCV infected children will have positive HCV RNA and 100% by 6 months if vertical transmission has occurred.^24,28,39,51,53^ The undetectable viral load on two occasions 3 months apart within 1 year excludes MTCT whereas two times HCV RNA positivity confirms the infection.^54^ Additionally, LFT should also be evaluated along with an HCV antibody test at 18 months of age, if deranged LFT is found despite negative antibody test, further evaluation with HCV RNA should be performed at 3 years of age and also in previous HCV RNA positive before 12 months of age.^53^

## TREATMENT OPTIONS FOR HEPATITIS C INFECTION

The available treatment options for the management of HCV infection (Table 1).

**Table 1:** Available medication for the treatment of hepatitis C virus infection.

Drugs regimens	HCV genotype indication	Use in Pregnancy
Interferon:		
(1)PegInterferon- alfa-2a ± (2)Ribavirin^*^	Genotype 1-6	(1)C, (2)X
(1)PegInterferon- alfa-2b ± (2)Ribavirin^*^	Genotype 1-6	(1)C, (2)X
Directly acting antivirals: DAA^†^ + PEG interferon combination: (1)Boceprevir+(2)Peginterferon (1)Telaprevir+(2)PEG Interferon (1)Simeprevirα+ (2)PEG interferon (1) Sofosbuvirα+ (2)PEG interferon	Genotype 1 Genotype 1 Genotype 1 Genotype 1 & 4	(1), (2)C (1)N/A, (2)C (1)C, (2)C (1)B, (2)C
DAA only: SOF containing regimens		
(1)Sofosbuvirα ± (2)Ribavirin^*^ (1)Sofosbuvirα + (2)Simeprevirα (1)Sofosbuvirα + (2)Ledipasvirα (1)Sofosbuvirα + (2)Daclatasvir (1)Sofosbuvirα + (2)Velpataspavirα (1)Sofosbuvirα + (2)velpatasvirα + (3)voxilaprevirα	Genotype 2 & 3 Genotype 1 Genotype 1, 4, 5, & 6 Genotype 1-6 Genotype 1-6 Genotype 1-6	(1)B, (2)X (1)B, (2)C (1)B, (2)B (1)B, (2)N/A^‡^ (1)B, (2)N/A (1)B, (2)N/A, (3)N/A
Non-SOF agents (1)Ombitasvirα + (2)Paritaprevirα + (3)ritonavirα + (4)dasabuvirα (1)Ombitasvirα + (2)Paritaprevirα + (3)ritonavirα (1)Elbasvirα + (2)grazoprevirα (1)Glecaprevirα + (2)Pibrentasvirα (1)Daclatasvir + (2)asunaprevir	Genotype 1 Genotype 4 Genotype 1 Genotype 1-6 Genotype 1 with renal impairment	(1)B, (2)B, (3)N/A, (4)B (1)B, (2)B,(3)N/A (1)N/A, (2)N/A (1)N/A, (2)N/A (1)N/A, (2)N/A

* Ribavirin is contraindicated due to its teratogenicity (FDA recommendation: X in pregnancy) and removed in other regimens which are given with/without ribavirin.

† αFDA approved

‡ Not available.

However, all those options cannot be considered during pregnancy due to the fetal complications risk, especially ribavirin is associated with fetal teratogenicity. Till date, none of the treatment options for hepatitis C during pregnancy are established in literature or recommended by any guidelines. However, treatment options could be considered in an individualized case basis to reduce the aforementioned obstetric and neonatal complications, particularly reducing vertical transmission either in single or co-infection with HIV/HBV infection. This reduction might limit the viral reservoir, prevent complications associated with chronic HCV infection in the offspring of the affected women and women herself, and reduce health care costs. Despite lesser benefits of HBV treatment in pregnancy, providers are considering HBV treatment in the third trimester or during pregnancy although there might be potential risks of drug exposure to the fetus. Furthermore, it doesn't provide a cure rather leads to the potential risk of disease flare-up following discontinuation of HBV therapy in the postpartum period.^41^ In the case of HCV/HIV coinfection, HIV coinfected women are treated as HIV mono-infection; however, treatment of HCV infected women would reduce the MTCT. A study conducted in the United States revealed 60% of HCV infected pregnant women are willing to get treatment if it benefited or reduced the vertical transmission, whereas only 21% willing to get DAAs therapy for self-cure even if it didn't affect MTCT.^49,55^

In general, HCV infected children above 12 years of age are normally treated; however, recent guidelines recommended DAAs regimens after 3 years of age. Before 3 years of age, there are chances of spontaneous resolution.^24,56^

**8.1 Interferon alpha with/without ribavirin**

The United States Food and Drug Administration (FDA) classified the first pharmacological formulation of IFN-α in pregnancy as category C since the molecule had an abortifacient effect in animals(rhesus monkeys) during the early/middle fetal period of organogenesis and late fetal development.^57^ IFN-alfa is used for the treatment of myeloproliferative diseases and chronic viral illnesses. Because the agent has antiproliferative activity, its potential adverse effects on a fetus are of big concern. Therefore, the use of IFN-α during pregnancy is greatly individualized even if clinically indicated. Furthermore, should an inadvertent administration of IFN-α during pregnancy occur, findings seem to encourage such women to continue the pregnancy. However, it is important to continue to monitor children who are exposed to IFN-α in utero.^58^ Although, there are case reports reporting use of interferon and ribavirin preconception or during pregnancy without congenital anomalies/birth defects, these drugs are contraindicated or categorized as category X during pregnancy by the FDA. The development of highly effective direct-acting antivirals (DAAs) have altered the prospects of curative therapy for people with HCV infection, cure rates of 90-100% has been reported with the currently available agents.^24,38,59^

The FDA classified ribavirin in Pregnancy Category X because of its embryocidal and teratogenic effects in animals. The fetal malformations reported in animal studies include abnormalities of the skull, palate, eye, jaw, limbs, skeleton, and gastrointestinal tract. Therefore, ribavirin is contraindicated for both HCV-infected childbearing women and HCV-infected male partners of any reproductive age group women, unless they take effective contraceptive measures.^12,24,32,44^ Besides,ribavirin-inducedspermatogenicabnormalities (cell toxicity, mutagenicity, and a decreased epididymal sperm count) reverted only 4-8 months after treatment withdrawal in all animal species studied. Therefore, women are advised to avoid pregnancy for at least 6 months after completion of ribavirin treatment for their male partners.^24,32,57^

**8.2 Directly acting antivirals**

DAAs regimens can be categorized as i) Sofosbuvir based regimens (single or in combination), and ii) non-sofosbuvir based regimens as mentioned in Table 1. Treatment of HCV with DAAs during pregnancy and breastfeeding is currently not recommended due to lack of data on safety, leaving HCV infected pregnant women untreated until after delivery, which in itself maybe distressing for the mother and deter her from breastfeeding.^60^ Given the lack of human studies, no DAA has yet been approved for use in pregnancy or during breastfeeding. Thus far, the safety of DAAs has not been evaluated in pregnant women, but animal studies have not demonstrated fetal risk. Consequently, we have reviewed the features of the DAAs approved for the treatment of chronic HCV infection in adults in the attempt to identify the most promising candidates, in terms of pharmacokinetic profile and adverse effects, for use in pregnancy or during breastfeeding.

**8.2.1 Sofosbuvir alone**

Sofosbuvir (SOF) appears to have a favorable pharmacokinetic profile and animal studies indicate that it may be safe during pregnancy. Thus, RBV-free SOF regimens, maybe the drug of choice for childbearing HCV infected women. The animal studies on pregnant rats and rabbits revealed no safety concerns regarding antenatal administration.^38,41,57^ A recent study appraised a 6 weeks SOF alone during pregnancy, followed by postpartum use of SOF and velpatasvir for 6 wks in HCV/HIV coinfected young females, revealed no HCV infection on the neonate and no abnormality/defect.^61^

In the case of children, SOF with ribavirin is the only FDA approved regimen for HCV GT 2 and 3. It can be considered for children ≥3 years of age with a weight-based regimen of both the drugs; however, duration of treatment varies according to the GT 2 and 3 of 12 and 24 weeks, respectively.^24,56,62^

**8.2.2 Sofosbuvir + ledipasvir**

An open-label, phase I study of pregnant women with GT-1 HCV infection and their infants, once-daily dose of SOF/ledipasvir for 12 wks at 23 and 24 weeks of gestation revealed safe and effective without a clinically meaningful difference in drugs exposure among pregnant versus non-pregnant women.^31,59,63^ Moreover, in the early study, a combination of SOF/LED stared in 28 weeks of gestation led to a cure in 8 HCV infected pregnant women.^37^ Most DAAs reported to cross the placenta and secreted in breast milk in animal model studies; still, most DAAs combinations showed a favorable safety profile. There is a rapid viral decline after treatment initiation. Therefore, treatment can be started at the end of the second trimester or early third trimester to avoid a critical period of organogenesis.^38,60,64^ Ledipasvir has a highly favorable pharmacokinetic profile and safe in animal embryos and fetuses as well. Moreover, SOF and ledipasvir combination, in particular, didn't show any fetal harm in animal studies, resulting in its FDA approval as pregnancy category B. Consequently, this combination formulation appears to be a good option in HCV infected women of child-bearing potential.^8,24,44,57,64^

In children, it can be considered at or above 3 years of age in genotype 1, 4, 5, or 6 with or without compensated cirrhosis. The dose varies with the weight of children (weight- <17kg, 17-35kg, and >35kg).^24,56,65,66^

**8.2.3 Sofosbuvir + Simeprevir**

Simeprevir was associated with reduced fetal weight, in utero fetal losses, early maternal deaths with high exposures, with teratogenic effects in the fetal skeletal system in animal studies. Moreover, it is excreted in the milk of lactating animals. So FDA classified it in category C and is not suitable for use in pregnant and breastfeeding mothers.^44,57^

**8.2.4 Sofosbuvir + Daclatasvir**

Daclatasvir, based on its pharmacokinetic profile, appears to have a wide safetymargin when used at therapeutic levels. But the dosages may have to be increased in pregnant women and were found to cross the placenta and exert a teratogenic effect in animals. Though it has been approved for marketing in the USA, It is still awaiting FDA pregnancy categorization.^38,57^ This regimen is recommended by AASLD HCV guidance 2018 for the treatment of adolescents and all children older than 3 years of age.^67,68^ A study on accidental exposure of this regimen during the time of conception reported no adverse birth outcomes although all women discontinued therapy early before nine weeks of gestation. Still, daclatasvir has demonstrated embryofetal toxicity in rabbits and rats.^60^ A phase III trial of this regimen in children aged 8-18 years or weight ≥17kg, revealed SVR12 of 98% with only mild side effects.^56,69^

**8.2.5 Sofosbuvir + velpatasvir**

This regimen is recommended for the treatment of pan-genotypic (1-6) HCV infection, has an SVR of 99%.^20,24,70,71^ No adequate human data are available to establish whether or not, this combination poses a risk to pregnancy outcomes. In animal reproduction studies, no evidence of adverse developmental outcomes was observed with the components (sofosbuvir or velpatasvir) at exposures greater than those in humans at the recommended human dose.^38,72^ This regimen has been approved and has no fetal toxicities in preclinical studies except when the dose was high enough to produce maternal toxicity as well.^41^ Though pregnancy categorization hasn't been assigned by FDA yet, it appears a safe option. Recently, a clinical trial is under evaluation but therapy started only in postpartum after cessation of breastfeeding.^73^ An ongoing clinical trial (NCT04382404 ) of single-arm, open-label, phase-1 study will be evaluating 12 weeks course of SOF/VEL in 10 HCV infected pregnant women during their second trimester. The study objectives are to reduce the risk of drug exposure during organogenesis, ensuring treatment completion by delivery, and reducing the risk of perinatal transmission. The neonatal outcomes will be evaluated at birth, 8 wks, 6 months, and 1 year.^74^ Another ongoing clinical trial (NCT03057847), a phase IV trial, SOF/VEL regimen for 12 weeks will be evaluated in 2 wks postpartum chronically HCV infected pregnant women with opioid use disorder. This study aims to assess the feasibility, acceptability, and adherence to this regimen at 4, 8, and 12 wks therapy, and will be followed for 15 months after posttreatment completion.^75^ This regimen is expected to have FDA approval for children aged 3-11 years on weight-based dosing.^76^ Moreover, this regimen can be considered in HIV-HCV coinfection as well.^20^

**8.2.6 Sofosbuvir + Velpatasvir + Voxilaprevir**

This regimen is recommended by American and European guidelines, has significant benefits in treatment-experienced patients or treatment failure cases, recommended for 12 weeks, and has significant SVR12 >95%. This regimen can be used in HIV-HCV coinfection; however, one has to be cautious about drug interactions with antiretroviral medications.^20,24,51,56^ Exposure to Voxilaprevir during pregnancy demonstrated no adverse embryofetal effects in rats and rabbits, and no data is available on placental transfer in animals.^60^

**8.3 Non-SOF combination regimens**

**8.3.1 Ombitasvir, Paritaprevir, Ritonavir, and Dasabuvir**

Viekirax® is a combination formulation composed of three pharmacologically active substances, namely Ombitasvir, Paritaprevir, and Ritonavir (Viekirax®). It is indicated only in combination with Ribavirin and/or Dasabuvir for the treatment of chronic hepatitis C infection in adults.^20,24^ Though it is classified as pregnancy category B by FDA, given its indication for use in combination with Ribavirin, it would not be suitable for women of childbearing potential. Combination with Dasabuvir is supposed to be relatively safe in pregnancy based on its pharmacokinetic profile and animal studies.^38,57^

**8.3.2 Elbasvir + grazoprevir**

This regimen is recommended for GT 1, 4, and 6 HCV infections, has SVR12 more than 90%.^20,24^ In animal reproduction studies, no evidence of adverse developmental outcomes were observed with its components (elbasvir or grazoprevir) at exposures larger than those in humans at the recommended human dose. FDA has not assigned this combination to any pregnancy categorization.^38,60,77^ However, phase III clinical trial revealed its efficacy on GT-1 and 4, and safety in case of chronic kidney disease cases.^78-80^ There is no need for dose adjustment in chronic kidney disease patients including a patient on hemodialysis.^20^

**8.3.3 Glecaprevir + Pibrentasvir**

Glecaprevir+ Pibrentasvir (Mavyret) regimen has a pan-genotypic effect, recommended for HCV GT-1-6, has efficacy of >95% with SVR8, and approved by FDA as well in 2019. Efficacy of this regimen is also supported by a recent meta-analysis.^20,24,27,81-83^ Generally, it is recommended in adolescents weighing ≥45kg or age ≥12 years with any GT with or without compensated cirrhosis.^24,56^ It can be considered in prior exposure to interferon-based SOF regimen with or without ribavirin, and without or with exposure to NS3/4A or NS5A protease inhibitors; however, duration of treatment is extended to 12 and 16 weeks in later two prior exposure cases with or without cirrhosis.^24,84-88^

In children, weight-based dosing of this regimen is expected to lead to FDA approval in children aged 3-11years in a recent trial.^24,89^ No adequate human clinical data of this combination are available regarding risk to pregnancy outcomes. But in animal reproduction studies, no adverse developmental effects have been observed at exposures greater than those in humans at the recommended human dose. This combination has been assigned AU TGA pregnancy category B1, while FDA categorization hasn't been assigned.^38,60,90^ Besides, it has shown promising efficacy and safety regarding chronic kidney disease cases.^79^ However, it is not recommended with other HIV protease inhibitors and contraindicated with atazanavir-containing regimens.^20^

**8.3.4 Daclatasvir + asunaprevir**

This combination was the first all-oral, interferon- and ribavirin-free regimen approved in Japan for treatment of patients with chronic HCV genotype 1 infection, including those with compensated cirrhosis.^91^ No clinical data is available on the safety of this combination in pregnant humans, but the sponsor has proposed pregnancy category B for Asunaprevir and, AU TGA Category B3 for a combination of Daclatasvir and Asunaprevir.^92^This combination therapy is highly effective and safe in patients with renal dysfunction.^93^

## HCV/HIV/HBV COINFECTION

All the coinfected HIV/HCV pregnant and postpartum women should be treated with HAART to reduce the HCV MTCT. However, no specific therapy is recommended for HCV infection during pregnancy except for the suppression of HIV replication.^32^Therefore, there is no difference in treatment in HCV pregnant women with/without HIV coinfection.^32^

The global prevalence of HBV/HCV coinfection is estimated at 5-10% and 1.4% in the United States only. This coinfection increased the risk of disease progression, decompensation, and the development of HCC.^56^ Therefore, treatment of one or both virus plays a prime role reducing the disease progression along with periodic retesting of HBV DNA and HCV RNA levels during and after initiation of therapy, especially when only one of the virus is being treated at a time.^24^ In HIV/HCV coinfection, previous studies demonstrated that PegIFN/RBVnotonly reduced liver-related complications but also HIV progression and mortality in coinfection. In the recent DAAs era, sofosbuvir/velpatasvir, sofosbuvir/daclatasvir, sofosbuvir/ledipasvir, pibrentasvir/glecaprevir, and elbasvir/grazoprevir have significant benefits treating HCV infection with SVR >95%.^56^

## VACCINATION AND PREVENTION

There is no recommended immunoglobulin for postexposure prophylaxis. A few vaccines are being explored and are under phase I and II clinical trials; however, none of them are recommended for the prevention of HCV infection till date.^22,39,94^ However, those HCV infected persons should be vaccinated for hepatitis A and B to prevent co-infection. Further, all HCV infected cases should be screened for HIV infection. In chronic liver disease or cirrhotic patients, the pneumococcal vaccine should be recommended.^17,27,39^

In conclusion, HCV infection is a major global health problem, more concerned during pregnancy or reproductive age group because treatment of this age group has a huge significance potential of reducing not only MTCT but also curing mother herself and diseasing burden in children associated with vertical transmission. Though no guidelines or recommendation are available till date for the use of any DAAs during pregnancy, individualized consideration of pan-genotypic DAAs regimens in the 2^nd^ and early 3^rd^ trimester of pregnancy would attenuate the MTCT rate, ultimately resulting in a reduction of HCV prevalence. This would help to meet the WHO target to reduce the disease burden by 2030.
